# The complete mitochondrial genome of *Acrossocheilus wenchowensis* (Cyprinidae, Barbinae) from Xinanjiang River

**DOI:** 10.1080/23802359.2019.1667916

**Published:** 2019-09-23

**Authors:** Tingshuang Pan, He Jiang, Yuting Hu, Jun Ling, Guoqing Duan

**Affiliations:** a Fisheries Institute, Anhui Academy of Agricultural Sciences, Hefei, China;; b Anhui Province Key Laboratory of Aquaculture & Stock Enhancement, Hefei, China;; c Huangshan Dingxin Ecological Agriculture Development Limited Company, Huangshan, China

**Keywords:** *Acrossocheilus wenchowensis*, mitochondrial genome, gene

## Abstract

The complete mt genome sequence of *Acrossocheilus wenchowensis* was obtained by PCR, containing 37 genes with 13 protein coding genes, 22 transfer RNAs (tRNAs), two ribosomal RNAs (rRNAs) and a non-coding control region.

Morphological classification of *Acrossocheilus* fishes is relatively difficult due to various kinds and similar shape. In this study, the complete mt genome of *Acrossocheilus wenchowensis* was sequenced.

*Acrossocheilus wenchowensis* was captured from upstream of Xinanjiang River, Anhui Province (29°36′28″N, 118°11′33″E). Voucher specimen (SF20180628-7) was deposited in Fish Specimen Library at Fisheries Institute Anhui Academy of Agriculture Sciences, Anhui, China. We amplified mitochondrial DNA using PCR method as reported (Ai et al. 2013). The complete mitochondrial genome of *A. wenchowensis* is 16,593 bp (MN266873).

The genome of *A. wenchowensis* are encoded on two strands and in two directions. The genome contains a total of 37 genes, including 13 protein-coding genes, 22 transfer RNAs and 2 ribosomal RNAs. The 13 protein-coding genes in the mt genome of *A. wenchowensis* also share features in start and stop codons with those in other *Acrossocheilus* fishes (Ai et al. 2013; Chen et al. 2015; Han et al. 2015; Xie et al. 2016).

Phylogenetic analysis was performed using 13 protein-coding genes by Neighbor-Joining (NJ). The clade containing *Acrossocheilus*, *Onychostoma*, *Puntius*, *Spinibarbus*, *Sinocyclocheilus* is well revealed with high supporting value in the phylogenetic tree (Figure 1).

**Figure 1.**
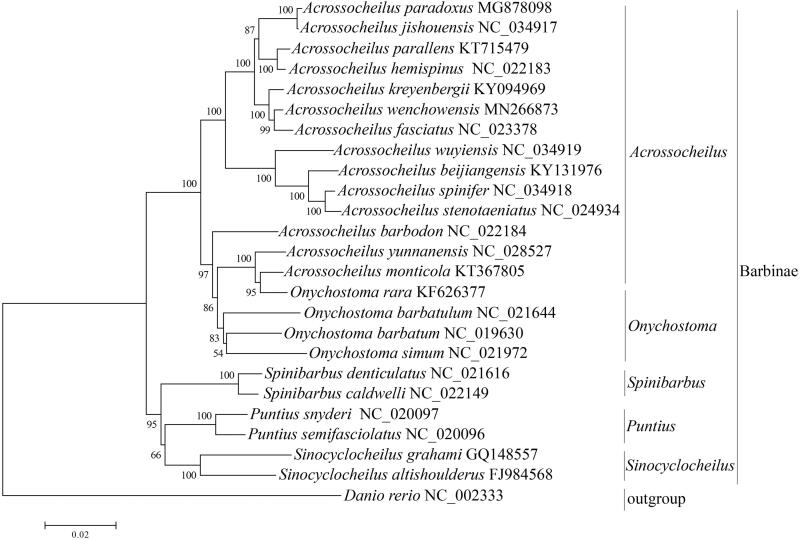
Neighbor-Joining phylogenetic tree inferred from amino acid sequence dataset of 13 protein-coding genes in Barbinae. The tree shows the topology based on concatenated data of 13 mitochondrial encoded protein sequences. Reconstruction was performed by MEGA X (64-bit).
